# Supplementary material for: A Structure Variation in qPH8.2 Detrimentally Affects Plant Architecture and Yield in Rice
Source: Plants (Basel). 2023 Sep 21;12(18):3336. doi: 10.3390/plants12183336 (PMC10536775; doi:10.3390/plants12183336)
Supplement: Supplementary file 1 [file plants-12-03336-s001.zip › Table S1 Single marker analysis in F2 population.pdf]

Table S1. Single marker analysis of plant height in F_2 population

Marker	Chr.	Position	*P*-Value
01C131	Chr1	41,979,406	4.27E-01
03C041	Chr3	9,409,200	6.81E-02
RM5556	Chr8	4,583,190	7.50E-06
M9060	Chr8	9,060,149	4.61E-55
M1508	Chr8	15,084,166	6.17E-41
10C051	Chr10	19,901,191	1.42E-01
