# Supplementary material for: A Structure Variation in qPH8.2 Detrimentally Affects Plant Architecture and Yield in Rice
Source: Plants (Basel). 2023 Sep 21;12(18):3336. doi: 10.3390/plants12183336 (PMC10536775; doi:10.3390/plants12183336)
Supplement: Supplementary file 1 [file plants-12-03336-s001.zip › Table S2 The primers used in this study.pdf]

Table S2. The primers used in this study

Primers	Sequence (5'-3')
RM5556	Forward: GTAAGCCATTTGCACGGACAAGG
	Reverse: GAGCTCAGGATCATCCCTACATGC
M5602	Forward: ACCTAACGAAACCAAAGA
	Reverse: TCGAGCAGGGTATCCAAT
M8061	Forward: TAATCACTCACCGCGTCACA
	Reverse: TGTTGACGATGTTCCAAGGC
M9060	Forward: GCCATATGCGGTGTTCTTTT
	Reverse: AGCGCATATCATGTTCAGTTCG
M1077	Forward: ACCATTAAATTACGGCCAGCC
	Reverse: CCTCTCAATTGACTGATTACGGT
M1103	Forward: TGAGCTATACTTGTTTGGAGAGAA
	Reverse: AGAGGATGGCTGAATGGTGA
M1307	Forward: GCTACAATCCGTTTGCCGAT
	Reverse: AAGGTGGACAGTCATTCTTCAA
M1508	Forward: TGTAGTGGGAAGGGAAAAAAACA
	Reverse: TGTGAACAGACTTCAAACGCT
M9204	Forward: GCATGCTCATTTGCTGGGAT
	Reverse: TCCCCCAAATAAACCCACTCT
M9392	Forward: CGATGACGTCTTTGCCGATT
	Reverse: GCTAGAGGTCGGTGTTGCTA
M9556	Forward: CAAGCTGGTCATGTGTGCAT
	Reverse: AAGGCGATTGCACAAACTGA
M9211	Forward: GTGTGGGATGACATGGGTCT
	Reverse: CAAAACGACGCAGCTGGTAA
M9212	Forward:CAGGTGGACCCCTTAAGTGG
	Reverse: AAGGATGGGGGTGTAGGAGA
M9285	Forward: CTTTTCCCTGCCTGCTTTCC
	Reverse: AGAGAAAAACACAAATCCCACTGA
M9322	Forward: TGCCTTTGATTGTGTAACCTTTG
	Reverse: TACCAACCAGAGCAACCACT
01C131	Forward: GTACTGCTAGCTTCATCCGT
	Reverse: AGTCATTCTAGCATTTCCGA
03C041	Forward: TTTTATGGGTTGAGGGTAGA
	Reverse: AACGTATTTGAGCTTGGCT
10C051	Forward: TTGATTATTATGCAGGAGCA
	Reverse: CGGATTTCCTGTACATGTTT
