# Supplementary material for: A Structure Variation in qPH8.2 Detrimentally Affects Plant Architecture and Yield in Rice
Source: Plants (Basel). 2023 Sep 21;12(18):3336. doi: 10.3390/plants12183336 (PMC10536775; doi:10.3390/plants12183336)
Supplement: Supplementary file 1 [file plants-12-03336-s001.zip › Table S3.pdf]

Table S3. The presence of a 42-kb insertion in different subgroups of rice

Variety Name	Genotype	Subgroup
Nipponbare	presence	Temperate japonica
Qiutianxiaoting	presence	Temperate japonica
Kongyu 131	presence	Temperate japonica
Qingjinzaosheng	presence	Temperate japonica
MAEKJO::IRGC 77666-1	presence	Temperate japonica
MR 19	presence	Temperate japonica
91-382::IRGC 63464-1	presence	Temperate japonica
Jinyuan 85	presence	Temperate japonica
Zhonghua 11	presence	Temperate japonica
Daohuangxiang	presence	Temperate japonica
MALAGKIT (PINELIPE)::IRGC 67444-1	absence	Tropical japonica
BUKU::IRGC 28683-2	absence	Tropical japonica
SAHULO FACHE SOYO::IRGC 66630-1	absence	Tropical japonica
SAL BUI BAO	absence	Tropical japonica
VARIRANGAHY::IRGC 69897-1	absence	Tropical japonica
American Huangkedao	absence	Tropical japonica
Azucena	absence	Tropical japonica
KETAN NANGKA::IRGC 19961-2	absence	Tropical japonica
ARC 11768::IRGC 21630-1	absence	Tropical japonica
MAK BOUAP::IRGC 30106-3	absence	Tropical japonica
PLE LIA::IRGC 73562-1	absence	Tropical japonica
SHANGYIPA::IRGC 64928-1	absence	Tropical japonica
SOM::IRGC 92221-1	absence	Tropical japonica

KAUK PAHLING::IRGC 95823-2	absence	Tropical japonica
CHAO MEO::IRGC 80273-1	absence	Tropical japonica
IH PEN SHIM MING::IRGC 26067-1	absence	Indica
Kahamu	absence	Indica
TAICHUNGNATIVE1	absence	Indica
Guangluai 4	absence	Indica
Tek Si Chut	absence	Indica
Zhenshan 97	absence	Indica
MUKKALA BAZAL::IRGC 77279-1	absence	Indica
Cisadane	absence	Indica
ITA 117::IRGC 75235-1	absence	Indica
MBEIMBEIHUN::IRGC 69730-1	absence	Indica
IRGA 318-11-6-9-2B::IRGC 117339-1	absence	Indica
WAS 173-B-B-6-2-2::C1	absence	Indica
KHAO GRADOOK CHAHNG::IRGC 17111-1	absence	Indica
X 21	absence	Indica
Budda	absence	Indica
Chorofa	absence	Indica
PSBRC 80	absence	Indica
IR64	absence	Indica
PR 106::IRGC 53418-1	absence	Indica
9311	absence	Indica
DERAWA::IRGC 64106-1	absence	Indica
HERATH BANDA::IRGC 67630-1	absence	Indica
LALKA (LAL DHAN)::IRGC 64946-1	absence	Indica
ZINYA KOLAMBA::IRGC 52402-1	absence	Indica

BR 116-3B-53::IRGC 39559-2	absence	Indica
LUDI GOCHYA::IRGC 26504-2	absence	Indica
OR 117-8::IRGC 39680-2	absence	Indica
GOBOL SAIL (BALAM)::IRGC 26624-2	absence	Indica
LARHA MUGAD::IRGC 52339-1	absence	Indica
AC 13 (T 141)::IRGC 5456-1	absence	Indica
BUCAYAB::IRGC 44357-1	absence	Indica
LUO SI ZHAN::IRGC 67211-1	absence	Indica
PHAN PHAE::IRGC 29652-2	absence	Indica
Tun Sart	absence	Indica
Khao Dawk Mali 105	absence	Indica
E 21::IRGC 33929-2	absence	Indica
GAM PERNG::IRGC 62135-1	absence	Indica
LIMA::IRGC 81487-1	absence	Indica
KHAO YAI GUANG::IRGC 65972-1	absence	Indica
ARC 11777::IRGC 21639-1	absence	Aus
JHONA 101::IRGC 27976-1	absence	Aus
HODARAWALA::IRGC 67631-1	absence	Aus
DV 86::IRGC 8840-1	absence	Aus
AMAKOYALI::IRGC 60878-1	absence	Aus
HIJOL DIGA::IRGC 31655-1	absence	Aus
KASALATH	absence	Aus
NA TEL BORO::IRGC 34749-1	absence	Aus
KASHA::IRGC 83865-1	absence	Basmati
ARC 18578::IRGC 42459-2	absence	Basmati
Binam	absence	Basmati

Karnal Local	absence	Basmati
JADO::IRGC 61966-1	absence	Basmati
Basmati 334	absence	Basmati
ARC 10497::IRGC 12485-1	absence	Basmati
wild12	absence	O.rufipogon
wild65	absence	O.rufipogon
wild111	absence	O.rufipogon
wild131	absence	O.rufipogon
wild219	absence	O.rufipogon
wild273	absence	O.rufipogon
DY159	absence	O.rufipogon
