# Supplementary figures and images for: A Structure Variation in qPH8.2 Detrimentally Affects Plant Architecture and Yield in Rice
Source: Plants (Basel). 2023 Sep 21;12(18):3336. doi: 10.3390/plants12183336 (PMC10536775; doi:10.3390/plants12183336)

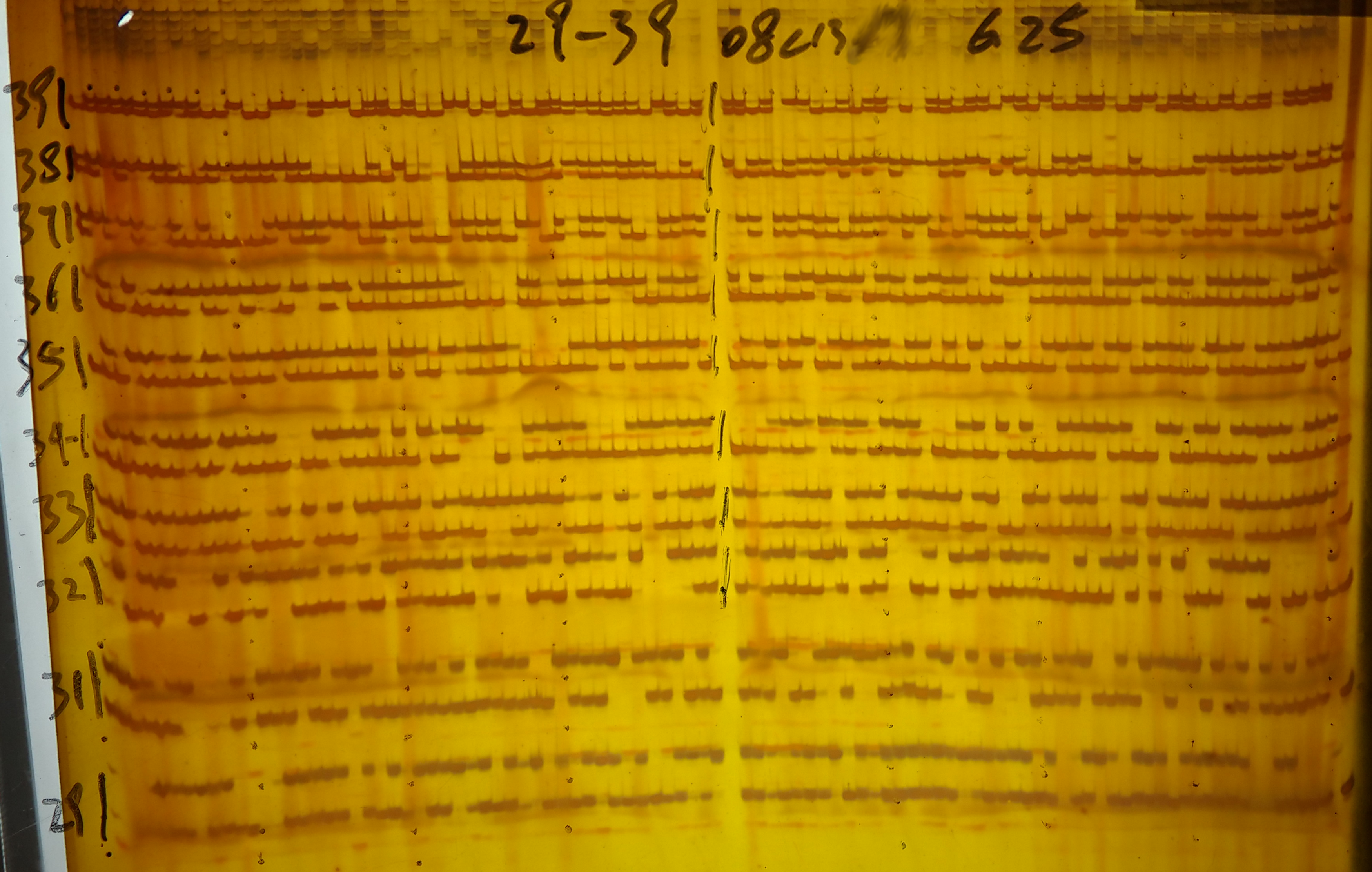

Supplement: Supplementary file 1 [file plants-12-03336-s001.zip › Figure S1 An electrophoregram.tif]
